# From adolescence to adulthood: validation of the levels of personality functioning questionnaire in a Turkish adult sample

**DOI:** 10.3389/fpsyt.2026.1776691

**Published:** 2026-03-26

**Authors:** Sefa Cosgun, Selim Arpacioglu, Suleyman Cakiroglu

**Affiliations:** 1 Private Practice, Istanbul, Türkiye; 2 Department of Psychiatry, Altınbas University, Istanbul, Türkiye; 3 Department of Child and Adolescent Psychiatry, Altınbas University, Istanbul, Türkiye

**Keywords:** adulthood, AMPD, disorder, functioning, LPFS, personality

## Abstract

**Introduction:**

The Levels of Personality Functioning Questionnaire (LoPF-Q) is a self-report tool developed to assess impairments in personality functioning, informed by Criterion A of the DSM-5 Alternative Model for Personality Disorders (AMPD) and the ICD-11 dimensional framework. The LoPF-Q 12–18 represents the adolescent version of the LoPF-Q Adult and has demonstrated robust psychometric properties in Turkish adolescent samples; however, the psychometric performance of the adult version in Turkish populations and its structural comparability across age groups remain underexplored.

**Methods:**

The current study evaluated the reliability, validity, and structural characteristics of the Turkish LoPF-Q Adult in a combined sample of 283 community adults and 65 clinical participants. Internal consistency was assessed via Cronbach’s alpha. Convergent and discriminant validity were examined through associations with the Brief Symptom Inventory (BSI). Clinical validity was evaluated by comparing LoPF-Q scores between the community group and a subset of patients diagnosed with personality disorders (n = 34). Additionally, bifactor modeling and receiver operating characteristic (ROC) analyses were conducted to examine factor structure and discriminative ability.

**Results:**

The LoPF-Q Adult demonstrated excellent internal consistency (Cronbach’s α =96). Strong correlations with general psychopathology on the BSI supported convergent validity, with distinct subscale-level patterns consistent with theoretical expectations. Clinical comparisons revealed significantly higher LoPF-Q total scores in individuals with personality disorders than in the community sample, with a large effect size (d = 1.56). Bifactor indices indicated a dominant general factor capturing the majority of shared variance, aligning with prior adolescent validation studies. ROC analysis yielded an area under the curve of.85, with an optimal cut-off score that provided balanced sensitivity and specificity.

**Discussion:**

These findings support the reliability and clinical utility of the Turkish LoPF-Q Adult as a dimensional measure of personality functioning severity. The strong general factor, robust discrimination between clinical and non-clinical groups indicates that the instrument captures structural impairment central to contemporary AMPD and ICD-11 models. Together with prior adolescent findings, the results are consistent with cross-age structural compatibility in the assessment of personality functioning rather than direct evidence of developmental continuity, while remaining preliminary pending longitudinal and invariance studies.

## Introduction

Over the last decade, the conceptualization of personality disorders (PDs) has shifted from a purely categorical paradigm to a dimensional perspective. In the fifth edition of the Diagnostic and Statistical Manual of Mental Disorders (DSM-5), an Alternative Model for Personality Disorders (AMPD) was proposed in Section III to address the limitations of traditional categorical diagnoses (1, 2). Central to this model is Criterion A, which defines PDs in terms of impairments in personality functioning – specifically in the self-domain (disturbances in identity and self-direction) and the interpersonal domain (disturbances in empathy and intimacy). In this dimensional framework, personality pathology is conceptualized as a continuum of severity of impairment in these core domains, rather than as distinct all-or-none categories. The shift inherent in Criterion A is profound, fundamentally refocusing the diagnosis on “what it means to be human,” positing that adaptive self- and interpersonal capacities are the core requirement for fulfilling adult life tasks (3). Notably, Criterion A is considered the “core” of personality pathology, capturing the fundamental impairment that distinguishes PDs from other psychopathology, while Criterion B describes the particular maladaptive trait manifestations of that pathology (3–5), a distinction that has been empirically supported by research demonstrating that Criterion A captures the core severity of personality pathology independent of specific traits (6). The 11th revision of the International Classification of Diseases (ICD-11) has adopted a highly similar approach: it reconceptualizes PDs as disorders of quantitatively varying severity of disturbed self and interpersonal functioning, doing away with most specific PD categories in favor of a single dimension of personality dysfunction (7–9). Both DSM-5 AMPD and ICD-11 frameworks therefore converge on the idea that impairments in personality functioning (Criterion A) represent the essence of PD pathology, providing a unifying severity dimension across different presentations (10).

Earlier DSM editions conceptualized personality disorders as discrete categories, an approach widely criticized for high diagnostic comorbidity, substantial within-category heterogeneity, and reliance on arbitrary diagnostic thresholds (11–13). Dimensional approaches address these limitations by conceptualizing personality pathology as a continuum of severity, allowing for more nuanced characterization of impairment and change over time. Early theoretical models emphasized that the severity of personality dysfunction represents one of the most robust predictors of current and future psychosocial impairment (4, 14). By explicitly incorporating a severity-based assessment of personality functioning (Criterion A), both DSM-5 AMPD and ICD-11 enable clearer differentiation between personality pathology and other forms of psychopathology based on level of dysfunction rather than symptom counts alone (15).

From a measurement perspective, however, the developmental nature of personality functioning introduces a methodological challenge: constructs may be developmentally continuous while their measurement properties are not. An instrument developed for adolescents cannot be assumed to function equivalently in adulthood without empirical verification, even when item content is preserved. Establishing adult validity is therefore not merely an extension of prior findings but a prerequisite for meaningful cross-age comparisons. This issue becomes particularly relevant for instruments designed to operate across age groups using identical item content.

To operationalize Criterion A (level of personality functioning) for clinical and research use, several assessment tools have been developed in recent years (16). The standard clinician-rated instrument in the DSM-5 AMPD is the Level of Personality Functioning Scale (LPFS), which evaluates self and interpersonal functioning on a 5-point rating from “no impairment” to “extreme impairment” (APA, 2013). Building on this, several self-report measures have been created to allow individuals to self-assess their personality functioning. These include the LPFS–Self-Report (17) and the LPFS–Brief Form 2.0 (18). Overall, these tools have demonstrated good reliability and validity in adult populations (19, 20). A notable limitation is that most existing instruments for personality functioning were designed either for adults or for adolescents, but not both. This divide hampers developmental continuity in research and practice, since it prevents the tracking of personality functioning from adolescence into adulthood using the same metric (21). The feasibility of extending adult measures to youth has been demonstrated, with the LPFS-BF 2.0 showing good psychometric properties in adolescent samples in recent studies (22, 23). However, this “downward extension” approach risks missing the unique developmental sensitivity of personality functioning; specifically, the crucial period in adolescence where the components of personality functioning bind together (coalesce) to form a coherent intrapsychic system (24). Given that PD pathology often has its onset in adolescence, there is a pressing need for instruments developed with a “bottom-up” perspective to capture these formative dynamics.

One measure that was specifically developed with developmental considerations in mind is the Levels of Personality Functioning Questionnaire 12–18 (LoPF-Q 12–18) (25). The LoPF-Q 12–18 is a self-report questionnaire designed to assess DSM-5 Criterion A in adolescents aged 12 to 18 years. The instrument was developed using a developmentally attuned approach, ensuring item content and wording were appropriate and sensitive to adolescent life contexts, unlike instruments merely adapted from adult forms (25). The original form consisted of 97 items and yielded a total score as well as domain scores for Identity, Self-Direction, Empathy, and Intimacy. Empirical studies have demonstrated strong psychometric properties of the LoPF-Q 12–18. It showed excellent internal consistency (e.g., total scale Cronbach’s α around.95) and clear clinical validity by robustly distinguishing adolescents with personality pathology from those without (25). Subsequent research has consistently supported a largely unidimensional structure for the LoPF-Q, indicating that a strong General Impairment Factor underlies the four domains (26, 27). Beyond the original German and English versions, the LoPF-Q 12–18 has been translated and validated across multiple languages and cultures, including Spanish, Lithuanian, and Turkish, among others, attesting to its cross-cultural robustness (6, 27, 28).

Despite the success of the LoPF-Q 12–18, a gap remained in extending its developmental sensitivity across the lifespan. The length of the 97-item form limited clinical feasibility, leading to the creation of shorter versions. Zimmermann and colleagues (2022) developed a 20-item screener using advanced statistical algorithms (29). More recent work refined a 36-item short-form structure now used across three psychometrically equivalent versions: LoPF-Q 12–18 Short (adolescent self-report), LoPF-Q 6–18 PR (parent-report), and LoPF-Q Adult (adult self-report) (30). All three versions share the same foundational items and four-domain structure with minimal wording adjustments to ensure age appropriateness. The aim of this standardized structure is to enable assessment using comparable metrics across age groups (31), allowing the stable core of personality pathology (Criterion A) to be measured consistently across development even as specific symptom expressions vary (heterotypic continuity) (24).

The LoPF-Q Adult represents a direct adaptation of the adolescent instrument based on the optimized 36-item set. By preserving the original item content with minimal age-neutral phrasing, it allows uninterrupted measurement of the same Criterion A constructs across age groups. This need is particularly evident in cultural contexts such as Turkey, where the LoPF-Q 12–18 has already been validated for adolescents (27), but the adult self-report form has not yet been validated, preventing empirical evaluation of cross-age comparability in this setting. Establishing validity in an adult Turkish sample therefore fills an important gap and contributes to the broader cross-cultural evidence base for Criterion A measures.

However, the psychometric properties of the LoPF-Q Adult have not yet been examined in a Turkish adult sample. The present study aims to evaluate the psychometric properties of the LoPF-Q Adult in a Turkish adult sample. Specifically, we aim to:

1. Examine internal consistency and factorial validity of the LoPF-Q Adult – determining whether the scale (and its subdomains) demonstrate high reliability and whether the underlying factor structure aligns with the expected construct (e.g., a largely unidimensional structure as found in the adolescent forms).
2. Evaluate convergent and discriminant validity by examining the relationship between LoPF-Q scores and a measure of general psychopathology, the Brief Symptom Inventory (BSI). We expect the LoPF-Q’s total impairment score to correlate strongly with overall symptom distress (convergent validity), while also displaying a distinct pattern of associations across BSI subscales that reflects the specificity of personality functioning impairment (discriminant aspects).
3. Assess clinical (known-groups) validity by comparing LoPF-Q scores between a community sample and a clinical sample of individuals diagnosed with personality disorders. We hypothesize that the PD group will show substantially higher LoPF-Q impairment scores than the non-PD group, consistent with prior findings in adolescent samples, thus demonstrating the questionnaire’s ability to discriminate between those with and without personality pathology.

By addressing these aims, our study seeks to validate the use of the LoPF-Q in an adult population and thereby bridge the assessment gap between adolescence and adulthood. This work will not only provide insight into the structure and extent of personality functioning impairments in adults but will also contribute to cross-cultural validation efforts for Criterion A measures. Ultimately, extending the LoPF-Q into adulthood advances the development of lifespan-compatible tools for PD assessment and provides a basis for future research on the developmental course of personality functioning impairments. The present study focuses on psychometric validation; developmental interpretations are considered exploratory and require longitudinal verification.

## Method

### Adaptation of the LoPF-Q 12–18 for adult use

After obtaining permission from the original developers, item wording was reviewed by a multidisciplinary expert panel (psychiatry, Turkish and English linguistics) for adult applicability. Cognitive interviews were conducted with 15 adults to assess clarity. One item was rephrased from “most people my age” to “most of my peers”; all remaining items were unchanged. The resulting instrument is referred to as the LoPF-Q Adult.

### Sample and procedure

The present study included a community sample and a clinical sample, with the clinical group further subdivided into participants with and without a personality disorder diagnosis. Clinical and community data were collected between January 2021 and June 2021.

### Community sample

Data were collected using the SoSci Survey platform. Automated quality checks (response time, inconsistent responding, attention indices) were applied. The Turkish scales were uploaded to SoSci Survey as a questionnaire, which was shared on various social platforms. Before completing the survey, participants were informed about the study, which was approved by the Ethics Committee of Medeniyet University (Approval No. 2020/0760, Approval Date: 16.12.2020), and about the voluntary nature of their participation. After submitting the informed consent form, participants required approximately twenty minutes to complete the questionnaire. Forms missing more than 10% of the data, participants with 12 points or above on the Non-Content-Based Responding Scale, and 19 responses flagged by the parameters for issues with data quality were excluded. The final community sample consisted of 284 people.

The participants of the community sample were chosen to fall between the ages of 18 and 61 (mean = 33.3, SD = 10.3). The minimum age criterion for inclusion was 18 years (legal adulthood). The composition of the community sample was as follows: 66.2% were women, 73.2% were university graduates, 49.3% were married, and 54.9% reported having an average income. Finally, 70.4% of participants reported no personal history of psychiatric disorders (**Table 1**).

**Table 1:** Socio-demographic characteristics.

		Community sample N=283 %	Clinic sample N=65 %	Total sample N=348 %
Age		18-61 / Mean 33.3 / SD 10.3	18-68 / Mean 33.3 / SD 11.1	18-68 / Mean 33.3 / SD 10.4
Sex		Male 33.8/ Female 66.2	Male 38.5/ Female 61.5	Male 34.7/ Female 65.3
Education	Primary School	2.9	10,8	4.3
	Secondary School	2.8	16.9	5.4
	High School	21.1	36.9	24.1
	University	73.2	35.4	66.2
Income	Low	10.6	24.6	13.2
	Medium	54.9	61.5	56.2
	High	34.5	13.8	30.7
Marital Status	Married	49.3	38.5	47.3
	Single	42.2	58.5	45.0
	Divorced	8.5	3.1	7.7
Have a child	Yes	44.8	38.5	43.6
	No	55.2	61.5	56.4
Psychiatric History	Present	29.6	70.7	37.2
	Absent	70.4	29.2	62.8

### Clinical sample

The clinical sample consisted of 65 patients who presented at the psychiatry clinic of a university hospital for the first time. Participants were not under psychopharmacological or psychotherapeutic treatment. Patients accepted to participate in the study were required to be at least 18 years of age, possess sufficient language and cognitive skills, have no neurodevelopmental disorders, and not be currently experiencing a mood or psychotic episode. All patients underwent a structured diagnostic interview (SCID-II) to establish categorical clinical grouping. The interview was used solely to define categorical clinical groups for known-groups comparisons and not as a criterion measure for validating the dimensional construct assessed by the LoPF-Q Adult. On this basis, 52% of participants in the clinical sample were diagnosed with a PD (n = 34), of those 55% were diagnosed with borderline personality disorder (BPD). Diagnoses of non–personality disorder conditions in the clinical sample were established through clinical interviews conducted by experienced psychiatrists. The Brief Symptom Inventory (BSI) was administered solely to assess the severity of general psychopathological symptoms and to characterize the clinical profile of the sample. Among the 31 non-PD participants, 20 were diagnosed with an anxiety disorder, seven with major depressive disorder, two with adjustment disorder, and two with obsessive-compulsive disorder. Patients in the clinical sample ranged from 18 to 68 years old (mean = 33.3, SD = 10.4). Of the participants in the clinical sample, 61.5% were women, 36.9% were university graduates, 58.5% were single, 61.5% had an average income, and 70.7% had a psychiatric history (**Table 1**).

### Measurements

In the present study, the term *personality disorder (PD)* refers to categorical DSM-based diagnoses used solely for clinical group classification. The term *personality pathology* denotes dimensional manifestations of maladaptive personality functioning. Impairment in personality functioning specifically refers to Criterion A — disturbances in self and interpersonal functioning — which constitutes the construct assessed by the LoPF-Q Adult.

### Sociodemographic form

The researchers created the sociodemographic form to examine participants’ sociodemographic characteristics (age, gender, educational background, level of income, and marital status).

### Levels of Personality Functioning Questionnaire Adult

The Levels of Personality Functioning Questionnaire (LoPF-Q) is a self-report instrument developed to assess impairments in personality functioning in line with Criterion A of the DSM-5 Alternative Model for Personality Disorders (AMPD) and the ICD-11 dimensional framework. The LoPF-Q 12–18 represents the adolescent version of the scale and was specifically designed for individuals aged 12 years and older, using developmentally appropriate and clearly formulated items (25). The revised adult version, the LoPF-Q Adult, retains identical item content with minimal wording adaptation, with only minor wording modifications to ensure suitability for adult populations.

Both versions assess self-functioning (identity, self-direction) and interpersonal functioning (empathy, intimacy), capturing core impairments in personality functioning. Although inspired by the DSM-5 Levels of Personality Functioning Scale (LPFS), the LoPF-Q follows a distinct developmental trajectory, resulting in an eight-domain structure rather than the twelve facets of the LPFS.

The Turkish version of the LoPF-Q 12–18 has demonstrated strong psychometric properties in adolescent samples (27), with high internal consistency (Cronbach’s alpha =95 for the total scale and ranging from.82 to.91 across subscales). The scale consists of 97 items rated on a five-point Likert-type scale (0 = strongly disagree to 4 = strongly agree), with higher total scores indicating greater impairment in personality functioning.

### The brief symptom inventory

The Brief Symptom Inventory (BSI) was developed by Derogatis as a shortened version of the Symptom Checklist-90 (SCL-90) to assess the severity of psychological symptoms across multiple domains. The BSI was adapted to Turkish culture by Şahin et al. (32, 33). In the original Turkish validation studies, internal consistency was examined in both non-clinical university student samples and clinical samples, yielding Cronbach’s alpha of.94 for the total scale and values ranging from.71 to.85 across subscales (33). In the present study, internal consistency was similarly high (Cronbach’s alpha =97) across the combined community and clinical participants.

### Semi-structured clinical interview for DSM-III-R personality disorders

The Structured Clinical Interview for DSM-III-R Personality Disorders (SCID-II) is a semi-structured diagnostic interview used to assess personality disorder diagnoses. The Turkish version of the SCID-II has demonstrated adequate validity and reliability (34). In the present study, the SCID-II was administered exclusively within the clinical sample to establish personality disorder diagnoses. SCID-II diagnoses were used as a categorical clinical comparator rather than as a validation criterion, because the LoPF-Q Adult operationalizes a dimensional construct of personality functioning (Criterion A) rather than categorical diagnosis membership.

Importantly, categorical PD diagnoses were used to anchor clinical grouping for known-groups comparisons and were not treated as a gold-standard criterion for validating the LoPF-Q Adult. The main aim of the study was the dimensional assessment of impairment in personality functioning (Criterion A), which was evaluated independently using the LoPF-Q Adult.

### Non-content-based responding scale

To assess participants’ attention, five additional items were included. Respondents indicated their agreement or disagreement with a five-point scale (1 = strongly disagree and 5 = strongly agree). Higher points show discordant answers. Consistent with prior administrations of this scale, participants with scores of 12 or greater were eliminated from the dataset (n = 12).

### Statistical analysis

All statistical analyses were performed using R statistical software (35). Initial descriptive examinations indicated that the data showed an acceptable level of normality for multivariate analyses, with skewness and kurtosis values remaining within the recommended ±1 range (36) Internal consistency was assessed using Cronbach’s alpha, and preliminary item analyses were conducted through corrected item–total correlations and descriptive indices to examine the quality of item functioning.

Confirmatory factor analyses (CFA) were then conducted on the full combined sample (community and clinical participants) to examine the latent structure of the LoPF-Q Adult. Because the items are ordinal and slightly non-normal, the Weighted Least Squares Mean and Variance adjusted (WLSMV) estimator was employed, as it provides robust parameter estimates and model fit indices under these conditions (37).

Competing theoretically plausible models were specified *a priori* to evaluate dimensionality. These models included a unidimensional structure representing a single overarching impairment in personality functioning, a correlated four-factor model reflecting the original domains of Identity, Self-Direction, Empathy, and Intimacy, and a bifactor model incorporating both a general personality functioning factor and four orthogonal domain-specific residual factors. Model fit was assessed using the Comparative Fit Index (CFI), Tucker–Lewis Index (TLI), the Root Mean Square Error of Approximation (RMSEA). Values of CFI and TLI of.95 or above, RMSEA values below.08 were taken as indicators of good model fit, following the recommendations of (38).

To further evaluate the construct validity of the LoPF-Q Adult and to determine the extent to which the measure reflects a general versus multidimensional personality functioning structure, model-based reliability and bifactor-specific psychometric indices were computed. Reliability estimates included total omega coefficients and hierarchical omega values, the latter quantifying the proportion of variance in total scores uniquely attributable to the general factor. Additional indices derived from the bifactor model provided a deeper understanding of dimensionality. The Explained Common Variance (ECV) quantified the proportion of shared variance across items accounted for by the general factor in comparison with the domain-specific factors. The Percentage of Uncontaminated Correlations (PUC) estimated the proportion of correlations driven exclusively by the general dimension, allowing for clearer interpretation of the scale’s unidimensional or multidimensional nature. Domain-level reliability estimates and item-level IECV values were used to examine whether specific domains retained unique and meaningful variance beyond the general factor. All factor loadings, reliability indices, and bifactor metrics were extracted and interpreted to assess the robustness of the measurement structure (39).

Convergent validity was examined by correlating LoPF-Q Adult total and domain scores with the Brief Symptom Inventory (BSI). Correlations were interpreted using conventional thresholds, with coefficients around.10 considered small, around.30 considered medium, and.50 or above considered large in magnitude (40). Clinical validity was evaluated by comparing community and clinical samples on LoPF-Q Adult scores and conducting Receiver Operating Characteristic (ROC) curve analyses to examine classification performance between predefined clinical groups. Effect sizes for group comparisons were calculated using Cohen’s d, defined as the mean difference between groups divided by the pooled standard deviation. Effect sizes were interpreted according to conventional benchmarks, with values of.20 indicating small,.50 medium, and.80 or greater large effects.

## Results

### Descriptive analysis and distribution

Sample characteristics are summarized in **Table 1**. Participants ranged in age from 18 to 68 years (M = 33.3, SD = 10.4), with females comprising 65.3% of the total sample. In the community sample, the majority of participants were university graduates (73.2%), whereas educational attainment was more evenly distributed in the clinical sample. A psychiatric history was reported by 70.7% of clinical participants and 29.6% of community participants. Within the clinical group, borderline personality disorder was the most frequent diagnosis (n = 19, 55%), while anxiety disorders were most common among non-PD patients (32%) (**Table 1**). No significant gender differences emerged in total or domain scores within the community sample.

### Reliability

Internal consistency of the LoPF-Q Adult was excellent for the total scale (Cronbach’s α =96; McDonald’s ω =97). Reliability for the primary domains was similarly high, with α values ranging from.85 to.92 and ω values ranging from.86 to.93. Secondary subscales demonstrated acceptable to good reliability (α =69–.88; ω =71–.89) (**Table 2**).

**Table 2:** Scale reliabilities Cronbach´s α and scale intercorrelation in the full sample N= 348 (N= 283 general population, N= 65 patients); clinical validity: significance p and effect size d of LoPF-Q Adult Turkish scale differences between general population and subsample of N=35 PD patients.

Scale	α	Intercorrelation				Clinical validity	
		ID	SD	EMP	INT	*p*	*d*
**LoPF-Q Total**	**.96**	.892	.884	.800	.788	.001	**1.56**
**Identity**	**.88**	–	.823	.570	.577	.001	1.30
Continuity	.79	.885	.684	.491	.584	.001	1.25
Coherence	.82	.922	.795	.537	.471	.001	1.07
**Self-Direction**	**.92**	.823	–	.544	.521	.001	1.31
Self-congruence	.84	.785	.949	.488	.521	.001	1.17
Purposefulness	.88	.790	.964	.548	.533	.001	1.35
**Empathy/Social Behaviour**	**.87**	.570	.544	–	.639	.001	1.04
Perspective Taking	.77	.550	.512	.840	.577	.001	0.92
Prosociality	.82	.479	.466	.928	.515	.001	0.89
**Intimacy / Attachment**	**.85**	.577	.521	.639	–	.001	1.34
Capacity for close relationships	.69	.530	.552	.532	.937	.001	1.05
Reciprocity	.81	.513	.415	.603	.788	.001	1.26

ID, Identity; SD, Self-Direction; EMP, Empathy; INT, Intimacy.

### Construct validity

Confirmatory factor analyses were conducted on the full combined sample (community and clinical participants) to evaluate the latent structure of the LoPF-Q Adult. Three competing models were tested: a unidimensional structure, a correlated four-factor model, and a bifactor model consisting of a general personality functioning factor and four orthogonal domain-specific factors. The unidimensional model demonstrated acceptable but comparatively weaker fit (CFI =950, TLI =947, RMSEA =070). The correlated four-factor model yielded slightly improved fit (CFI =959, TLI =955, RMSEA =064). Among all models, the bifactor model showed the best overall performance, with superior fit indices (CFI =967, TLI =963, RMSEA =059).

Inspection of standardized factor loadings revealed that most items loaded more strongly onto the general factor than onto the domain-specific residual factors. Loadings on the domain factors were comparatively lower and more variable across items.

Bifactor-based indices further described the distribution of variance across the model. The general factor accounted for 76% of the common variance (ECV =76), and its hierarchical omega coefficient was.80. Domain-specific omega values ranged from.07 to.20. The Percentage of Uncontaminated Correlations (PUC) was elevated, and item-level IECV values were predominantly above.70.

At the domain level, bifactor reliability and variance estimates showed that domain-specific omega coefficients (ωS) were.17 for Identity,.07 for Self-Direction,.17 for Empathy, and.20 for Intimacy. The proportion of common variance attributable to the general factor relative to each domain (ECV*_GS_*) was.82 for Identity,.86 for Self-Direction,.69 for Empathy, and.60 for Intimacy. Factor determinacy (H) values for the domain factors ranged from.36 to.58 (**Table 3**).

**Table 3:** Confirmatory factor analysis fit indices and bifactor indices.

		CFI		TLI	RMSEA
Confirmatory Factor Analysis					
One-Factor		.950		.947	.070
Four-Factor		.959		.955	.064
Bifactor		.967		.963	.059
Bifactor Model Psychometric Indices					
*General Factor Indices*					
ECV		OmegaH		PUC	
.76		.80		High	
Domain-Specific Indices (Residual Factors)					
	Identity	Self-Direction	Empathy	Intimacy	
ωS	.17	.07	.17	.20	
ECV*_GS_*	.82	.86	.69	.60	
H	.36	.42	.51	.58	

One-factor = unidimensional model; Four-factor = correlated four-factor model (Identity, Self-Direction, Empathy, Intimacy); Bifactor = general factor plus domain-specific factors. CFI = Comparative Fit Index; TLI = Tucker–Lewis Index; RMSEA = Root Mean Square Error of Approximation; ΩH = hierarchical omega; ωS = domain-specific omega; ECV = Explained Common Variance; ECV*_GS_* = domain-level explained common variance relative to the general factor; H = factor determinacy.

### Convergent, discriminant, and clinical validity

Convergent validity was supported by strong associations between LoPF-Q Adult scores and psychological distress measured by the Brief Symptom Inventory (**Table 4**). The total LoPF-Q Adult score correlated strongly with overall distress (r =76, p <.001), depressive symptoms (r =75, p <.001), and anxiety (r =69, p <.001). Strong associations were observed between LoPF-Q Adult domain scores and psychological distress, particularly for identity and self-direction (r =74–.78), whereas empathy and intimacy showed moderate correlations (r =36–.56) (**Table 4**).

**Table 4:** Intercorrelations between psychopathology and personality functioning.

	LoPF-Q, criterion A domains				
	Identity	Self-Direction	Empathy	Intimacy	Total
Brief Symptom Inventory					
Anxiety	.65	.70	.47	.44	**.69**
Depression	.75	.78	.47	.49	**.75**
Negative Self-Concept	.71	.75	.49	.50	**.74**
Somatization	.53	.58	.36	.36	**.55**
Hostility	.57	.63	.56	.44	**.66**
**Total**	**.72**	**.77**	**.51**	**.49**	**.76**

All correlations are significant at p <.001. Effect size: r >0.10 small, >0.30 medium, >0.50 strong.

Receiver operating characteristic (ROC) analysis was conducted to examine the ability of the LoPF-Q Adult to differentiate participants classified as having a personality disorder from community participants. The scale demonstrated significant discrimination (AUC =85, p <.001, 95% CI:.77,.93). The score maximizing the Youden index was 169 (sensitivity = 77%, specificity = 80%), representing a sample-specific classification threshold rather than a clinical diagnostic cut-off.

When LoPF-Q Adult scores were compared across the community sample and the clinical sample (PD and non-PD patients), a clear dimensional gradient emerged. The community sample showed the lowest levels of impairment, followed by the non-PD clinical group, and the highest impairment was observed among patients diagnosed with personality disorders, consistent with the theoretical framework of personality functioning and the dimensional approach.

Direct comparisons between community participants and PD patients (n = 34) indicated significantly higher total and subscale scores in the PD group, reflecting greater impairment in personality functioning. The effect size for the total score was large (d = 1.56), with similarly large effects observed across all domains (**Table 5**).

**Table 5:** Clinical validity differences in LoPF-Q scores and assigned significance p for a) Community sample vs No-PD patient sample and b) Community sample vs PD patient sample.

	Community population N= 283	No-PD patients N= 31		PD patients N=34		Effect size
	Mean (SD)	Mean (SD)	p Value	Mean (SD)	p Value	d
**LoPF-Q Total**	**130.3 (40.7)**	**156.5 (45.2)**	**.001**	**197.6 (45.3)**	**.001**	**1.56**
**Identity**	32.2 (12.1)	37.8 (13.2)	.016	48.8 (13.3)	.001	1.30
Continuity	14.1 (6.2)	17.5 (6.6)	.004	22.0 (6.4)	.001	1.25
Coherence	18.1 (7.4)	20.3 (8.0)	.115	26.8 (8.8)	.001	1.07
**Self-Direction**	37.5 (14.8)	50.9 (14.8)	.001	59.7 (18.8)	.001	1.31
Self-congruence	18.3 (7.1)	24.7 (8.0)	.001	27.8 (9.0)	001	1.17
Purposefulness	19.3 (8.5)	26.3 (8.1)	.001	32.0 (10.2)	.001	1.35
**Empathy/Social Behaviour**	29.0 (11.6)	31.6 (12.0)	.251	42.9 (14.8)	.001	1.04
Perspective Taking	11.8 (5.3)	13.3 (6.1)	.137	17.5 (6.9)	.001	0.92
Prosociality	17.3 (7.7)	18.3 (8.6)	.489	25.4 (10.3)	.001	0.89
**Intimacy /Attachment**	31.6 (10.9)	36.4 (13.5)	.023	46.1 (10.7)	.001	1.34
Capacity for close relat.	14.6 (5.0)	16.4 (6.0)	.033	19.7 (4.7)	.001	1.05
Reciprocity	17.0 (7.3)	20.2 (9.4)	.047	26.4 (7.6)	.001	1.26

effect size: d >0.20 small, >0.50 medium, >0.80 large.

Within the clinical sample, PD patients also demonstrated significantly higher total and domain scores than non-PD patients, with a large effect size for the total score (d =91) and comparable effects across subscales. Comparisons between non-PD clinical participants and community participants revealed statistically significant differences across most domains, whereas empathy showed a smaller effect size (d =65) (**Table 5**).

## Discussion

This study provides evidence that the psychometric properties of the LoPF-Q in a Turkish adult sample are highly consistent with the growing literature conceptualizing impairments in personality functioning as a single, pervasive dimension observable across age groups. The high internal consistency observed in both community and clinical groups, the emergence of a strong general factor in our structural analyses, and the markedly elevated scores among individuals diagnosed with personality disorders collectively indicate that personality functioning reflects a core impairment not reducible to categorical diagnoses and not reducible to categorical diagnostic boundaries. This pattern converges with previous findings on the adolescent version of the LoPF-Q, where studies in both international and Turkish samples have similarly reported an essentially unidimensional structure with a dominant general factor capturing overall severity of impairment (27). Observing the same structural organization in adulthood supports conceptual compatibility with lifespan models, although developmental continuity cannot be inferred from cross-sectional data (3, 41).

Consistent with prior adolescent findings, the present adult data show a comparable structural organization, with the general factor explaining most of the variance. This structural similarity aligns with studies linking impaired personality functioning in adolescence to later psychosocial difficulties, including emotion regulation problems, unstable interpersonal relationships, and broader psychiatric vulnerability—difficulties also characteristic of adults with elevated LoPF-Q scores (42, 43). Taken together, the adolescent and adult literature suggests that Criterion A may represent a structurally consistent vulnerability construct, emerging in adolescence and crystallizing more fully in adult personality organization. It should be noted, however, that this developmental continuity is inferred conceptually from converging findings across age groups, rather than empirically demonstrated through formal tests of measurement invariance between adolescent and adult versions of the LoPF-Q.

The coherence of this structure becomes even more apparent when considering the bifactor indices, which provide a detailed view of how variance is distributed across general and domain-specific factors. In our adult sample, the general factor accounted for the vast majority of shared variance, while the domain-specific residuals contributed modestly—an index pattern nearly identical to that seen in the English and Turkish validations of the LoPF-Q 12–18. Kerr et al. reported high ECV and OmegaH values for the adolescent measure, indicating that the scale functions as an essentially unidimensional measure despite its four-domain design. Similarly, our earlier Turkish adolescent study demonstrated that the general factor absorbed most of the common variance, with the domains acting as nuanced expressions of global dysfunction. Replicating this structure in adulthood suggests that the dominance of the general factor is not restricted to a specific age group but reflects a stable psychometric characteristic of the LoPF-Q framework (44, 45). This pattern is consistent with a hierarchical conceptualization of Criterion A in which identity, self-direction, empathy, and intimacy do not operate as independent dimensions but as differentiated expressions of a common structural impairment (46). The low domain-specific omega coefficients indicate that most reliable variance is captured by the general factor, supporting interpretation of the LoPF-Q Adult primarily as a severity indicator of personality functioning. Clinically, domain elevations may aid descriptive formulation but should not be interpreted as independent dimensions (13). Accordingly, the LoPF-Q Adult is best interpreted using a severity-first framework in which domain scores qualify — rather than constitute — the evaluation of personality functioning.

Beyond these psychometric results, our earlier validation work in adolescents and adults offers important cross-age comparative context. Using identical methodology across age groups, prior studies consistently showed that community samples exhibit lower levels of impairment in adulthood relative to adolescence, whereas individuals with clinically significant personality pathology show far less change across developmental periods (47–49). Although these cross-sectional comparisons were not part of the analyses reported here, they parallel longitudinal findings showing previously reported age-related differences in personality functioning alongside the relative stability of clinically relevant impairment (14, 50). Considered alongside the strong general factor found in the present study, these observations reinforce the view that the LoPF-Q captures a structurally coherent core of personality dysfunction and underscore the importance of examining personality functioning across the lifespan.

The strong group differences observed across community participants, non-PD clinical patients, and individuals diagnosed with personality disorders provide solid support for the clinical utility of the LoPF-Q Adult. Total and domain scores demonstrated a clear dimensional gradient, with progressively higher levels of impairment from the community sample to the non-PD clinical group and the PD group. In particular, large effect sizes between PD patients and community participants, as well as substantial differences between PD and non-PD clinical patients, indicate that the LoPF-Q Adult is sensitive to clinically meaningful variations in the severity of personality functioning. Importantly, these clinical differences are derived from a PD subgroup predominantly characterized by borderline personality pathology.

The interpretation of these group differences should also consider the composition of the community sample. Approximately 29.6% of participants reported a history of psychological or psychiatric treatment based on a single self-report item. This variable did not assess diagnosis, severity, or recency of treatment and therefore does not necessarily indicate the presence of a current mental disorder. Nevertheless, the inclusion of individuals with past treatment experience may have elevated mean impairment levels in the community group and reduced observed group differences. Accordingly, the community sample should not be interpreted as a healthy normative reference but as a population-based comparison group reflecting variability in functioning within the general population. From a measurement perspective, this composition likely reduces artificial separation between clinical and non-clinical groups and therefore provides a more conservative test of discriminative validity.

The strong discriminative performance of the LoPF-Q Adult further supports its clinical utility. ROC analyses indicated that total scores differentiated individuals diagnosed with personality disorder from community adults, yielding an AUC of.85 and sample-specific classification threshold of 169. However, this discriminative performance should be interpreted within the diagnostic framework used to define the clinical group. Because personality disorder diagnoses were established using DSM-III-R–based SCID-II criteria, the ROC findings reflect the instrument’s ability to distinguish individuals meeting categorical DSM-III-R PD diagnoses rather than severity thresholds defined within DSM-5 AMPD or ICD-11 models. Accordingly, the cut-off value should be considered preliminary and sample-specific pending replication in samples classified using dimensional diagnostic frameworks. Thus, the ROC results reflect correspondence with categorical diagnostic grouping rather than a dimensional severity threshold.

While sampling differences prevent developmental interpretation of absolute cut-off values, the overall pattern of strong group differentiation remains informative. Together with the large effect sizes observed between groups, the findings indicate that the LoPF-Q Adult captures clinically meaningful variation in structural impairment (7, 31).

From a diagnostic framework perspective, the clinical group was defined using a DSM-III-R–based categorical interview, whereas the LoPF-Q Adult is grounded in the dimensional AMPD/ICD-11 model. This creates a potential conceptual misalignment between the diagnostic classification and the construct being measured. Categorical diagnoses rely on symptom thresholds, whereas Criterion A reflects the severity of structural dysfunction. Consequently, bidirectional misclassification may occur: individuals with clinically significant impairment may not meet categorical thresholds, while some categorical diagnoses may not correspond to maximal personality functioning impairment. Importantly, such misclassification would be expected to attenuate rather than inflate group differences (51). Therefore, the large observed effect sizes likely represent conservative estimates of the instrument’s discriminative validity.

This interpretation is further supported by the diagnostic composition of the clinical subgroup. The predominance of borderline personality disorder in the clinical subgroup further supports this interpretation, as borderline pathology is strongly associated with severe disturbances in identity integration and interpersonal functioning — domains directly assessed by Criterion A (52). Accordingly, the findings are best interpreted as reflecting differences in structural severity rather than merely categorical diagnostic labels.

A central point of debate in the Criterion A literature concerns whether measures of personality functioning reflect impairments specific to personality pathology or broader psychological distress (53). In the present study, LoPF-Q Adult total scores showed strong associations with psychological distress as measured by the BSI—a pattern consistently reported across both adolescent and adult LoPF-Q research. At a methodological level, the use of self-report instruments to assess both personality functioning and symptom distress may contribute to shared method variance, potentially inflating the magnitude of these associations and raising valid concerns regarding discriminant validity (14, 54).

Conceptually, however, substantial overlap is theoretically expected (2). Impairments in identity, self-direction, empathy, and intimacy represent structural vulnerabilities that shape how individuals experience, regulate, and respond to emotional and interpersonal difficulties captured by broad symptom measures. Recent conceptual work further emphasizes that personality functioning should be distinguished from psychosocial functioning, which reflects downstream outcomes in social, occupational, and relational domains rather than the underlying organization of personality structure (23, 55).

Moreover, acute symptom states—particularly depressive affect—may introduce state effects that negatively bias individuals’ self-perceptions of functioning, further contributing to empirical overlap between these constructs (54). Importantly, prior studies demonstrate that even after accounting for general psychopathology, Criterion A measures continue to predict functional impairment, relational instability, and clinical severity, underscoring their incremental validity beyond transient symptom distress (56). Taken together, while shared variance should be interpreted cautiously, the present findings support the clinical and theoretical validity of the LoPF-Q as a structural indicator of pervasive personality dysfunction (14, 57–59).

Future research would benefit from employing multi-method assessment approaches, including clinician-rated measures and observational data, to further disentangle structural personality functioning from transient symptom distress and to more precisely evaluate the discriminant validity of Criterion A measures across developmental stages.

### Limitations and future directions

Several limitations of this study should be acknowledged. First, the cross-sectional design precludes conclusions about developmental trajectories or temporal stability of personality functioning. Although previous adolescent and adult findings suggest both mean-level change and moderate rank-order stability, longitudinal research is required to clarify developmental pathways across the lifespan.

Second, the clinical personality disorder (PD) subgroup was relatively small and diagnostically skewed, with a predominance of borderline personality disorder diagnoses. Although this distribution is common in clinical settings, it limits the generalizability of findings related to clinical discrimination across the full spectrum of personality disorders. Accordingly, the observed group differences may primarily reflect borderline personality pathology rather than PDs.

Third, PD diagnoses were based on the SCID-II using DSM-III-R criteria rather than more recent diagnostic instruments aligned with DSM-5 AMPD or ICD-11 frameworks. Although SCID-II represented the standardized diagnostic procedure available in the clinical setting at the time of data collection, this introduces a conceptual misalignment between categorical diagnosis and the dimensional construct assessed by the LoPF-Q Adult. Because categorical diagnoses rely on symptom thresholds whereas Criterion A reflects the severity of structural dysfunction, bidirectional misclassification may occur: some individuals with clinically significant impairment may not meet categorical criteria, while some categorical diagnoses may not correspond to maximal personality functioning impairment. Such misclassification would be expected to attenuate rather than inflate observed group differences. Consistent with this interpretation, the predominance of borderline personality disorder in the clinical subgroup is noteworthy, as borderline pathology is strongly associated with disturbances in identity integration and interpersonal functioning — the core domains assessed by Criterion A. Future studies employing updated dimensional diagnostic interviews will be important to further clarify the relationship between contemporary diagnostic systems and personality functioning assessment.

Fourth, although the LoPF-Q demonstrated strong psychometric performance, measurement invariance across developmental periods and cultures was not examined. Future longitudinal and multi-group invariance studies are therefore needed to empirically test the equivalence of the measurement structure across developmental stages.

In addition, the adult sample covered a broad age range (18–68 years), and analyses were conducted without stratifying adulthood into developmental subphases. Therefore, the results should be interpreted as supporting structural consistency across a broad adult age range rather than age-invariant measurement properties. Although prior research suggests moderate rank-order stability of personality functioning across adulthood (14), meaningful developmental changes in symptom expression and severity have also been documented across different phases of adult life (60, 61). In the absence of age-stratified analyses or age-related invariance testing, the extent to which the present findings generalize equally across younger, middle-aged, and older adults remains uncertain. Future studies employing age-stratified and invariance analyses will be required to determine whether identical score interpretations can be applied across early, middle, and late adulthood.

In addition, the community sample cannot be considered a strictly asymptomatic reference group. Approximately 29.6% of participants reported a history of psychological or psychiatric treatment based on a single self-report item, which did not assess diagnosis, severity, or recency of treatment. Although common in general population samples, the inclusion of individuals with past treatment experience may have elevated mean impairment levels in the community group and attenuated observed group differences. Consequently, normative interpretations should be understood as reflecting a general population comparison rather than a healthy control sample.

Finally, the sample showed a marked overrepresentation of female participants. This imbalance likely reflects recruitment characteristics and differential participation rates rather than intentional sampling bias. Nevertheless, given established gender differences in the prevalence and clinical expression of personality pathology (62, 63), this distribution may limit the generalizability of the findings. Future studies with more balanced gender representation will be important to examine potential gender-related differences in the assessment and manifestation of personality functioning impairment.

## Conclusion

In summary, this study provides robust evidence that the Turkish LoPF-Q Adult is a reliable and clinically meaningful measure of personality functioning. The strong general factor, high internal consistency, and clear differentiation between community participants and clinically characterized groups indicate that the scale captures the core severity dimension central to contemporary dimensional models of personality pathology.

Consistent with prior findings in adolescent samples, the present results support the interpretation of personality functioning as a largely unidimensional construct reflecting global impairment in self and interpersonal functioning. In this context, the total score appears to be the most psychometrically robust and clinically informative indicator, whereas domain scores may best be understood as nuanced expressions of overall severity rather than fully independent dimensions.

Together with earlier adolescent validation studies, the findings are consistent with the use of the LoPF-Q within a lifespan-oriented assessment framework compatible with DSM-5 AMPD and ICD-11 frameworks. While longitudinal and multi-method research is needed to further clarify developmental trajectories, measurement invariance, and incremental validity across adulthood, these results provide an important foundation for lifespan-oriented assessment of personality functioning in both research and clinical practice (64).

## Data Availability

The raw data supporting the conclusions of this article will be made available by the authors, without undue reservation.
