# Correction: From adolescence to adulthood: validation of the levels of personality functioning questionnaire in a Turkish adult sample

**DOI:** 10.3389/fpsyt.2026.1850195

**Published:** 2026-04-20

**Authors:** Sefa Cosgun, Selim Arpacioglu, Suleyman Cakiroglu

**Affiliations:** 1 Private Practice, Istanbul, Türkiye; 2 Department of Psychiatry, Altınbas University, Istanbul, Türkiye; 3 Department of Child and Adolescent Psychiatry, Altınbas University, Istanbul, Türkiye

**Keywords:** adulthood, AMPD, disorder, functioning, LPFS, personality

Author Sefa Cosgun was erroneously assigned to affiliation “Department of Child and Adolescent Psychiatry, Altınbas University, Istanbul, Türkiye”. This affiliation has now been removed for author Sefa Cosgun.

Affiliation “Private Practice, Istanbul, Türkiye” was omitted from the paper. This affiliation has now been added for author(s) Sefa Cosgun.

Author Selim Arpacioglu was erroneously assigned to affiliation “Department of Child and Adolescent Psychiatry, Altınbas University, Istanbul, Türkiye”. This affiliation has now been removed for author Selim Arpacioglu.

Author Suleyman Cakiroglu was erroneously assigned to affiliation “Department of Psychiatry, Altınbas University, Istanbul, Türkiye”. This affiliation has now been removed for author Suleyman Cakiroglu.

Affiliation “Department of Psychiatry, Altınbas University, Istanbul, Türkiye” was omitted for author Suleyman Cakiroglu. This affiliation has now been added for author(s) Suleyman Cakiroglu.

The original version of this article has been updated.

